# A comparative analysis of genomic and phenomic predictions of growth-related traits in 3-way coffee hybrids

**DOI:** 10.1093/g3journal/jkac170

**Published:** 2022-07-06

**Authors:** Alain J Mbebi, Jean-Christophe Breitler, Mélanie Bordeaux, Ronan Sulpice, Marcus McHale, Hao Tong, Lucile Toniutti, Jonny Alonso Castillo, Benoît Bertrand, Zoran Nikoloski

**Affiliations:** Bioinformatics Group, Institute of Biochemistry and Biology, University of Potsdam, Potsdam-Golm 14476, Germany; Systems Biology and Mathematical Modeling Group, Max Planck Institute of Molecular Plant Physiology, Potsdam-Golm 14476, Germany; Centre de Coopération Internationale en Recherche Agronomique pour le Développement, Montpellier 34398, France; Fundación Nicafrance, Finca La Cumplida Km. 147 Carretera Matagalpa - La Dalia, 3 Km al Noreste, Matagalpa, Nicaragua; National University Ireland Galway, Plant Systems Biology Laboratory, Ryan Institute, School of Natural Sciences, Galway H91 TK33, Ireland; National University Ireland Galway, Plant Systems Biology Laboratory, Ryan Institute, School of Natural Sciences, Galway H91 TK33, Ireland; Bioinformatics Group, Institute of Biochemistry and Biology, University of Potsdam, Potsdam-Golm 14476, Germany; Systems Biology and Mathematical Modeling Group, Max Planck Institute of Molecular Plant Physiology, Potsdam-Golm 14476, Germany; Center for Plant Systems Biology and Biotechnology, Plovdiv 4000, Bulgaria; Centre de Coopération Internationale en Recherche Agronomique pour le Développement, Montpellier 34398, France; Fundación Nicafrance, Finca La Cumplida Km. 147 Carretera Matagalpa - La Dalia, 3 Km al Noreste, Matagalpa, Nicaragua; Centre de Coopération Internationale en Recherche Agronomique pour le Développement, Montpellier 34398, France; Bioinformatics Group, Institute of Biochemistry and Biology, University of Potsdam, Potsdam-Golm 14476, Germany; Systems Biology and Mathematical Modeling Group, Max Planck Institute of Molecular Plant Physiology, Potsdam-Golm 14476, Germany; Center for Plant Systems Biology and Biotechnology, Plovdiv 4000, Bulgaria

**Keywords:** genomic prediction, phenomic prediction, 3-way coffee hybrids, chlorophyll *a* fluorescence, GenPred, Shared Data Resource

## Abstract

Genomic prediction has revolutionized crop breeding despite remaining issues of transferability of models to unseen environmental conditions and environments. Usage of endophenotypes rather than genomic markers leads to the possibility of building phenomic prediction models that can account, in part, for this challenge. Here, we compare and contrast genomic prediction and phenomic prediction models for 3 growth-related traits, namely, leaf count, tree height, and trunk diameter, from 2 coffee 3-way hybrid populations exposed to a series of treatment-inducing environmental conditions. The models are based on 7 different statistical methods built with genomic markers and ChlF data used as predictors. This comparative analysis demonstrates that the best-performing phenomic prediction models show higher predictability than the best genomic prediction models for the considered traits and environments in the vast majority of comparisons within 3-way hybrid populations. In addition, we show that phenomic prediction models are transferrable between conditions but to a lower extent between populations and we conclude that chlorophyll *a* fluorescence data can serve as alternative predictors in statistical models of coffee hybrid performance. Future directions will explore their combination with other endophenotypes to further improve the prediction of growth-related traits for crops.

## Introduction

Food production must increase by 60–70% by 2050 to feed the increasing world’s population. In parallel, climate change is expected to reduce the yields of key crops (Arora 2019). One way of addressing these challenges is by devising policies conducive to sustainable agricultural production, which competes for resources (e.g. arable land and water) with other industrial sectors. Another way that makes use of the growing phenotypic and genotypic data is to speed up the breeding of crop varieties (i.e. genotypes), which are resilient to environmental cues exacerbated by climate change (e.g. water availability, ambient temperature), while increasing the yield.

Before the era of genomic prediction (GP) (Meuwissen *et al.* 2001), the development of improved plant varieties has mostly relied on classical breeding whose implementation is limited due to the long selection cycles, high phenotyping costs, reduced reliability when dealing with low heritable traits, and sensitivity to environmental fluctuation (Tuberosa 2012). GP aims to overcome these limitations by combining genotypic data and phenotypic data of the training population through a predictive model that in turn is used to compute genomic estimated breeding value for individuals in a population with genotypic data but yet to be phenotyped (Poland *et al.* 2012). With the proliferation of cost-effective high-throughput genotyping platforms, GP is rapidly changing breeding perspectives in both crop (Jannink *et al.* 2010; Heslot *et al.* 2015) and animal (Goddard and Hayes 2007; Hayes *et al.* 2009) breeding.

Furthermore, the genetic evaluation in animal breeding when full pedigree and genomic information are combined (Dou *et al.* 2017) and when some genotypes are missing (Christensen and Lund 2010) paved the way for their application in crop breeding. In the latter and for low heritable traits, it has been shown that combining pedigree information and single-nucleotide polymorphism (SNP)-based relationships in a kinship matrix can improve the predictability of GP models (Velazco *et al.* 2019). However, classic estimators for genetic relatedness using molecular markers are less effective for low-coverage sequencing data, which often exhibit high levels of genotype uncertainty and missing data (Dou *et al.* 2017); moreover, access to high-quality reference genomes still remains a challenge for several species (e.g. polyploid species).

Effective growth and performance evaluation using noninvasive methods has been identified as one of the key challenges in plant and crop improvement programs (Baker and Rosenqvist 2004). High phenotyping costs and developmental delays to the emergence of important traits in perennial crops, such as coffee, justify the popularity of GP. In the quest for alternatives to genotyping, using endophenotypes as predictors has been recently proposed and used (Fernandez *et al.* 2016; Guo *et al.* 2016; Schrag *et al.* 2018). The resulting findings suggest that phenomic prediction (PP), based on the availability of phenotypes used as predictors in the training and testing population, may be a suitable alternative to GP.

Chlorophyll *a* fluorescence (ChlF) has been routinely used for many years to noninvasively monitor the photosynthetic performance of plants (Baker 2008) and to evaluate plant tolerance to abiotic stressors (Stirbet *et al.* 2018). In a recent study (Gamboa-Becerra *et al.* 2021), the effectiveness of this technique in assessing the physiological state of coffee plants subjected to a combination of biotic and abiotic stress has also been demonstrated. The observations that ChlF measurements can be used to estimate the operating quantum efficiency of electron transport in coffee leaves that directly relates to coffee plant health and oxidative stress level have led to the use of this trait in examining photosynthetic performance in contrasted field situations (Toniutti *et al.* 2017, 2019). Furthermore, near-infrared reflectance spectroscopy (NIRS) wavelength data on wheat grain and leaf tissues have been shown to result in PP models that outperform GP models (Rincent *et al.* 2018).

Because changes in fluorescence induced by the illumination of dark-adapted leaves are qualitatively correlated with changes in CO_2_ assimilation, under some circumstances, fluorescence emissions in photosynthetic organisms could be correlated to their photosynthetic rates (Stirbet *et al.* 2018). Using this approach, we hypothesize that ChlF transients can be employed in high-throughput screens for growth and vigor in coffee. The objectives of our study are to assess if there is a relation between the photosynthesis efficiency and the vigor/growth of coffee trees in different contexts that can potentially be used in breeding program. To this end, we make use of facile to obtain phenomic data (i.e. ChlF) and compare the performance of GP and PP for 3 growth-related traits from 2 three-way hybrid (H3W) coffee populations. The resulting models are used to understand the impact of conditions that mimic different coffee-growing contexts.

## Materials and methods

### H3W populations

Clonally propagated F1 hybrid “Centroamericano” (T.05296 × Rume Sudan, henceforth H1) plants were used as maternal donor in crosses with Ethiopian lines ET47 and Geisha 3, producing 2 segregating populations (H3W). Note that T.05296 (a Sarchimor cultivar) is known for its tolerance to coffee leaf rust disease obtained through introgression from the Timor Hybrid. T.05296 is also wind resistant, widely adaptable to varying altitudes and climates and has an exceptional root system enabling it to adapt to different types of soil.

### DNA extraction

DNA was extracted from leaf tissues of 8-month-old plants using DNeasy Plant kit (Qiagen). DNA quality was evaluated by Agilent 2100 Bioanalyzer High Sensitivity DNA assay (Agilent Technologies, Santa Clara, CA, USA) and quantified by Qubit 2.0 Fluorometer (Invitrogen, Carlsbad, CA, USA).

### Probe design

The 3 parental genotypes (i.e. ET47, Geisha, and H1) were first sequenced to identify polymorphic regions. Libraries were prepared using “Celero DNA-Seq” kit (NuGEN, San Carlos, CA, USA) per manufacturer’s instructions and quantified using the Qubit 2.0 Fluorometer. Sequencing was performed on an Illumina NovaSeq 6000 (Illumina, San Diego, CA, USA) in a paired-end 150 mode. Low-quality reads and adapter regions were removed using ERNE (2.2.1) (Del Fabbro *et al.* 2013) and Cutadapt v1.18 (–overlap 10 –time 2 –minimum-length 50 -mask-adapter) (Martin 2011). Reads were aligned using BWA-MEM (0.7.17) (Li and Durbin 2009) to a draft genome of Coffea arabica from a Caturra red cultivar (RHJU01) (Zimin *et al.* 2018). Variant calling was performed using GATK (4.1.0.0) (McKenna *et al.* 2010).

Sequencing resulted in >400-M reads for each parental genotype, supporting the identification of an 3,127,161 SNPs. Due to the allotetraploid genome of *Coffea arabica*, many of these were likely false positives. SNPs associated with repeat regions of *Coffea canephora* were first removed, resulting in 1,212,811 SNPs (Denoeud *et al.* 2014; Smit *et al.* 2013–2015). To further remove collapsed homeologous regions, a custom Perl script was used to retain only those which were homozygous in at least one of the 3 parental lines. Relative levels of heterozygosity for each variety in this remaining 260,015 SNPs reflected those anticipated, with 35,162 (14%), 32,150 (12%), and 219,479 (84%) heterozygous sites in ET47, Geisha, and H1, respectively.

For ET47 and Geisha, 18,514 heterozygous SNPs were selected with a minimum span of 50 bp. For H1, further examination was applied to identify regions with high numbers of SNPs that are likely to be regions of introgression from the ancestral rust tolerant Timor Hybrid variety. For putative introgressed regions, 35,274 SNPs were selected (minimum span 100 bp) and, for other regions, 32,838 SNPs were selected (minimum span 50 bp). A total of 86,626 SNPs were reduced to 80,584 when selecting for regions critical to probe design for single primer enrichment technology (SPET) (Scaglione *et al.* 2019). A total of 151,362 probes were designed for regions up- and down-stream of the target SNPs (NuGEN, Tecan Group).

### H3W genotyping

H3W populations from crosses between the F1 hybrid and each Ethiopian line (i.e. H1xET47 and H1xG) were then subjected to targeted sequencing for SNP genotyping. Libraries were prepared using the “Allegro Targeted Genotyping” protocol from NuGEN Technologies with the described probes and 100 ng/μL of DNA as input. Libraries were quantified using the Qubit 2.0 Fluorometer, and their size was checked using the High Sensitivity DNA assay from Bioanalyzer or the High Sensitivity DNA assay from Caliper LabChip GX (Caliper Life Sciences, Alameda, CA). Libraries were quantified through qPCR using the CFX96 Touch Real-Time PCR Detection System (Bio-Rad Laboratories, Hercules, CA) and sequenced on the Illumina NovaSeq 6000 in a 150-bp single-end mode. Low-quality reads and adapter regions were removed using ERNE (1.4.6) (Del Fabbro *et al.* 2013) and Cutadapt v1.18 (Martin 2011), both with default parameters. Reads were aligned using BWA-MEM (0.7.17) (Li and Durbin 2009) to RHJU01 (Zimin *et al.* 2018) and retained where mapping quality is >10. SNP calling was performed in GATK following best practices for germline short variant discovery (DePristo *et al.* 2011).

SNPs with smaller than 5% minimum allele frequency and call rate smaller than 95% were removed. In addition, we excluded all samples with more than 10% of missing genotypes and those without a match in the phenomic (i.e. ChlF) and phenotype data. Mean imputation of the missing values in the SNP data that passed the filtering rules was then performed, resulting in a final data with 74 and 119 samples for H1xET47 and H1xG populations, respectively, with altogether 61,950 markers.

### Field experiment

Each of the 2 segregating hybrid populations were cultivated at La Cumplida farm in Matagalpa region, Nicaragua (GPS coordinates 13.0008989–85.8514005). Plants were first grown in polypropylene cells containing 540 mL of a 70% mixture of blonde peat (PG-mix) and 30% sand, supplemented with 4 g/L of fertilizer (Multicote). After 45 days, each cell received 3 g of fertilizer (Multicote). At 10 months after sowing, plants were transferred to 5-gallon pots (height 0.37 m; width 0.32 m) and subsequently treated with 5 g/L of fertilizer (Multicote) every 4 months.

Immediately after transferring to 20-L pots, plants were moved into the first treatment condition shown in Table 1. Plants were first maintained under shade for a complete acclimation at altitude level 600 m where average daily high temperatures were 24°C. Then, they were transferred to full sun conditions for 2 months, followed by 3 months under shade and similar temperature conditions. Finally, the plants were transferred to full sun at an altitude of 1,300 m where average daily high temperatures were 20°C. Consistent shading to 50% was achieved by the use of an artificial shade net.

**Table 1.** Successive treatment conditions applied on the H3W coffee populations before their transfer to the field.

Treatment	Altitude	Temperature	Condition	Duration	Mimicking
1	600	23.6	Shade	3	n/a (acclimation)
2	600	24.5	Full sun	2	Open field
3	600	23.5	Shade	2.5	AFS established
4	1300	20	Full sun	2	Cooler temperatures

Altitude, duration, and temperature are, respectively, measured in meters, months, and °C. AFS denotes agroforestry system.

### Phenotypic data

At the end of each treatment, plants were phenotyped for several characteristics [e.g. trunk diameter (TD), height, total number of leaves, and ChlF]. The first measurement (i.e. after acclimation) took place when ET47, G, H1xET47, and H1xG were 13 months old, and the second and third measurements were, respectively, taken when the plants were 16 and 18 months old. For the 3 measurements, H1 parent clones were 4 months younger than the other genotypes due to differences in the plant production time.

### Phenomic measurements: ChlF

ChlF measurements were conducted between 2 and 4 AM with a Handy PEA chlorophyll fluorimeter (Handy-Plant Efficiency Analyser, Hansatech Instruments, Norfolk, UK) on mature leaves (L3). Every measurement was performed on apparently healthy, fully light-exposed leaves. Measurements were taken 5 times on each plant during 3 consecutive nights at the end of each treatment, resulting in 1,980 measurements per plant or 7,920 measurements in total for the analyzed populations. During night, leaves are dark adapted and when they are illuminated, ChlF intensity shows characteristic changes called fluorescence transient (Stirbet *et al.* 2018). ChlF transients were induced by 1-s illumination with an array of 6 light-emitting diodes providing a maximum light intensity of 3,000 photosynthetically active radiation. The fast fluorescence kinetics (from F0 to FM, where F0 and FM are, respectively, the minimum and maximum measured chlorophyll fluorescence of photosystem II in the dark-adapted state) were recorded from 10 μs to 1 s. For the analysis, 18 parameters (Supplementary Table 1) were selected as the most relevant to explain photosynthesis [i.e. IBR, PI total, phi(Ro), phi(Eo), psi(Eo), phi(Po), phi(Po)/(1-phi(Po)), dRo/(1-dRo), psi(Eo)/(1-psi(Eo)), RC/ABS, REo/RC, DIo/RC, ETo/RC, TRo/RC, ABS/RC, Fo, Fm, and Fv/Fm].

### Statistical methods

Throughout the text, the term “phenotypic” refers about the target traits (i.e. response variables) while the term “phenomic” refers to endophenotypes (i.e. predictors in the PP models). The comparative analysis is concerned with evaluating the performance of genomic and PPs on 3 growth-related traits [i.e. leaf count (LC), tree height (TH), and TD] under the following settings: setting S1 that aims to select the best-performing H3W family by comparing hybrids H1xET47 and H1xG based on the predictability of GP and PP models. Traits and phenomic data were constructed by concatenating the respective measurements over all treatment conditions after the acclimation period. Setting S2 contrasts the predictive abilities of GP and PP models in H1xET47 and H1xG under established agroforestry system (AFS) that corresponds to treatment 3 in Table 1. To this end, only traits and ChlF data of the corresponding treatment were considered. This setting also evaluates the effect of including more predictors in PP models. For this second goal, ChlF measurements were concatenated from treatments 2–4 while using the traits only from treatment 3. Setting S3 evaluates GP and PP models based on their abilities to predict traits in the next treatment condition. Specifically, we compare the predictive abilities of these models using the current environmental conditions for H1xET47 and H1xG as the training set and the successive conditions as the test one. Because the 2 hybrids have 1 parent in common (i.e. H1), we finally consider setting S4, where we train the models with data from 1 family and predict traits of the other one. Phenomic and traits data are constructed as in setting S1. For completeness, Supplementary Fig. 3 provides a graphical representation of data construction for each setting.

In what follows, we present the statistical models used in the comparative analysis and the details of the cross-validation strategy. Since in our case the number of markers is much larger than the number of observations, the following modeling approaches were used instead:

#### Ridge regression

The marker effects are estimated by solving the following optimization problem.

(1) $$\hat{b_{i}}(\text{RR})=\underset{b_{i}}{\text{argmin}}||y_{i}-Xb_{i}|{|}_{2}+\lambda ||b_{i}|{|}_{2},$$

where λ≥0 is a penalty parameter, estimated via cross-validation.

#### LASSO

Replacing the *L*_2_ norm by the *L*_1_ norm, the optimization problem in (1) becomes the least absolute shrinkage and selection operator (LASSO) (Tibshirani 1996) that simultaneously select variables and shrink coefficients by solving

(2) $${\hat{b}}_{i}(\text{LASSO})=\underset{b_{i}}{\text{argmin}}||y_{i}-Xb_{i}|{|}_{2}+\lambda ||b_{i}|{|}_{1}.$$

Equivalently $\hat{B}(\text{mLASSO})=\underset{B}{\text{argmin}}||Y-XB|{|}_{2}+\lambda ||B|{|}_{1}$ for multi-response.

#### Elastic net

To overcome some shortcomings of LASSO, such as SNPs in high linkage disequilibrium and lack of group selection, elastic net (EN) (Zou and Hastie 2005; Ogutu *et al.* 2012), an extension of LASSO can serve as a remedy. EN blends ridge regression (RR) and LASSO penalties and optimizes

(3) $${\hat{b}}_{i}(\text{EN})=\underset{b_{i}}{\text{argmin}}||y_{i}-Xb_{i}|{|}_{2}+\lambda_{1}||b_{i}|{|}_{2}+\lambda_{2}||b_{i}|{|}_{1}.$$

LASSO’s variable selection ability is preserved via the $L_{1}$ penalty in (3) and its $L_{2}$ counterpart enables group selection.

#### Genomic best linear unbiased predictor

Implemented in this study with the R-package BGLR (de los Campos and Pérez-Rodríguez 2014), genomic best linear unbiased predictor (GBLUP) was obtained considering the linear mixed effect model formulated as,

(4) y=Zu+ϵ.

The residual error *ϵ* is assumed to be normally distributed with zero-mean and $\text{var}(\varepsilon )=I\sigma_{\epsilon }^{2}$, with I the identity matrix of appropriate dimension. In this setting, Z represents the incidence matrix for individual effects and u is a vector of genotype random effects assumed to follow a multivariate Normal distribution with $\text{var}(Zu)=K\sigma_{u}^{2}$, where $\sigma_{u}^{2}$ is the genetic additive variance and $K=ZZ^{\prime }$ is the realized genomic relationship matrix.

#### Bayesian LASSO

Previously discussed GP methods assume common variance for all SNP effects. However, for some traits, departure from normality is often exhibited in practice and should be accounted for. Bayesian LASSO (BL) (Park and Casella 2008; de los Campos *et al.* 2009) allows to take such effects into account. It can be shown that Equation (2) is minimized when regression coefficients are assumed to be independently distributed with Laplace (i.e. double-exponential) priors (Hans 2009). With a product of *p* independent and zero-mean double-exponential densities as prior on $b_{i}$, BL solves

(5) $$p(y_{i}|b_{i},\sigma_{\epsilon }^{2})=\prod_{l=1}^{n}N(y_{il}|x_{il}^{\prime }b_{i},\sigma_{\epsilon }^{2}).$$

Using the scale-mixture parametrization and the hierarchical model (see Park and Casella 2008 for details), BL was implemented using the R package BLR (Pérez *et al.* 2010) with the hyperparameter as recommended in the package and using a chain of 20,000 iterations and a burn-in sample of 5,000 iterations. We would like to point out that 20,000 iterations were considered because beyond this number, no substantial change was observed on the predictability.

Unlike single trait, multiple-trait (MT) GP models combine information from individual lines and analyze MTs simultaneously. The potential of MT methods to improve predictive ability in GP has been proven (Jia and Jannink 2012; Lado *et al.* 2018; Budhlakoti *et al.* 2019). For completeness, the following 2 MT prediction methods are also included in the comparative analysis.

#### MT BayesB

Most MT GP models are built upon a restrictive assumption that a given locus affects simultaneously all the traits or none of them. To overcome this limitation, Cheng *et al.* (2018) used mixed priors to develop MT Bayesian regression methods allowing a locus to influence any combination of traits. Our comparative analysis focuses only on their MT BayesB (mBayesB), where vectors of marker effects are assumed to be multivariate normally distributed with mean zero and locus-specific covariance matrix having an inverse Wishart prior. Details regarding the derivation of full conditional distributions of parameters can be found in Cheng *et al.* (2018).

#### $L_{2,1}$ -norm regularized multivariate regression and covariance estimation

The $L_{2,1}$-norm regularized multivariate regression and covariance estimation (L21-joint) (Mbebi *et al.* 2021), models traits jointly by assuming that the response variables follow a multivariate Gaussian distribution with precision matrix Ω.

(6) $$f(B,\Omega )=\underset{B,\Omega }{\text{argmin}}\left\{J(B,\Omega )+\lambda_{1}||\Omega |{|}_{1}+\lambda_{2}||B|{|}_{2,1}\right\},$$

with tuning parameters $\lambda_{1}\geq 0$ and $\lambda_{2}\geq 0$ to be obtained from the data and

$$J(B,\Omega )=tr\left[\frac{1}{s}(Y^{\prime }-B^{\prime }X^{\prime })\Omega {\left(Y^{\prime }-B^{\prime }X^{\prime }\right)}^{\prime }\right]-\text{log}\;|\Omega |.$$

As shown in (6), the $L_{2,1}$ and $L_{1}$ losses are, respectively, applied on the marker effects and precision matrix to provide sparse estimates of the regression coefficients and the precision matrix using an iterative optimization procedure. At each iteration, the estimated Ω is used to refine the estimation of marker effect until convergence.

#### *K*-fold cross-validation and hyperparameters

Given the moderate sample size after data filtering, *n *=* *74 and *n *=* *119 for H1xET47 and H1xG, respectively, we perform *K*-fold cross-validation, randomly sampling individuals in phenotype, SNP and ChlF data sets into *K *=* *3 blocks of approximately equal size. We divided each data set into training and validation sets, composed of 2- and 1-fold, respectively. We used 2-fold to train the statistical models and the remaining fold for validation. We quantified model performances by the Pearson correlation coefficient between predicted and observed trait values in the validation set (Crossa *et al.* 2010; Ober *et al.* 2012). This was done until every fold was used as validation and the performance is then computed as the average value over the 3-fold (Zhou *et al.* 2017).

Nevertheless, some models in the comparative analysis have hyperparameters that need tuning (i.e. optimizing). To avoid using the same data to optimize model parameters and performance evaluation that often leads to overfit (Cawley and Talbot 2010), nested 3-fold cross-validation was used. This is accomplished by 2 loops and splitting the data into training, validation, and test sets. In the inner loop, each training set is used to fit the model and the hyperparameters are subsequently selected after evaluating the model on the validation set. In the outer loop, the independent test set is used to quantify the prediction abilities. For a better assessment, we then used re-sampling and repeated this procedure 20 times.

#### Heritability and genetic correlation

We next recall 2 of the most important genetic parameters to consider for breeding. (1) Heritability is defined as the proportion of phenotypic variance explained by underlying genetic effects (Falconer 1989). The broad-sense heritability is computed as $H^{2}=\frac{\sigma_{G}^{2}}{\sigma_{G}^{2}+\sigma_{E}^{2}+\sigma_{\text{GxE}}^{2}/e+\sigma_{\varepsilon }^{2}/e}$ (Hallauer *et al.* 2010), where *e* represents the number of environments (i.e. treatment conditions), $\sigma_{G}^{2},\;\sigma_{E}^{2},\;\sigma_{\text{GxE}}^{2}$, and $\sigma_{\varepsilon }^{2}$ are, respectively, the genetic, environment, genetic by environment, and residual components of the variance. The variance partition of each factor is estimated by fitting a linear mixed model with all above effects as random and fixed effects of intercept. The computations were implemented with the R package lme4 (Bates *et al.* 2015).

(2) Genetic correlation between trait *i* and *j* is defined as $r_{g}^{2}=\frac{\text{cov}(g_{i},g_{j})}{\sqrt{\text{var}(g_{i},g_{j}))}}$, where *g_i_* and *g_j_* are the genetic effect of trait *i* and *j*, respectively, and is equivalent to the Pearson correlation coefficients between their genetic effect (Galic *et al.* 2019). The genetic effects are obtained based on the SNP data using rrBLUP model for each trait and Pearson correlation coefficients between phenotypic traits (i.e. LC, TH, and TD) and ChlF parameters as the genetic correlations.

## Results and discussion

### Heritabilities and genetic correlations of the studied traits

First, we quantify heritability since it directly relates to the extent to which a given trait is predicted by genetic factors, and therefore can be improved by breeding. To this end, we partition the variance into environment (E), genetic (G), genetic by environment (G×E), and residual (*ε*) components. We considered phenotypic traits (i.e. LC, TH, and TD) as well as the 18 ChlF traits in each family and estimated their broad-sense heritability. Strong variability of traits heritability was exhibited (Supplementary Tables 2 and 3), with maximum values always observed for TH in both H1xG ($H^{2}=62\%$) and H1xET47 ($H^{2}=77\%$) families. Although some mild peak values of about (24%) could be observed, overall heritability for most ChlF traits was very small in both families. Further look at the GxE component of the phenotypic variance indicates a genetic component to the plasticity of these traits.

To further assess the usability of ChlF traits in PP models, we quantified the proportion of variance shared by 2 traits due to genetic effects using the genetic correlations (Galic *et al.* 2019) between phenotypic traits (i.e. LC, TH, and TD) and ChlF parameters. Since in this setting, a trait expressed in multiple environments is treated as a different trait, this lead to a 9 × 54 genetic correlation matrix (i.e. 3 and 18 traits for each treatment condition). Our findings (Supplementary Tables 4 and 5) show that in both families, the highest genetic correlations between ChlF and the target traits were achieved with ChlF parameters measured under the acclimation condition (e.g. IBR, $\Psi E_{o},\;\varphi E_{o}$). Furthermore, the high variability of genetic correlation observed between treatment conditions could indicate that in line with the large GxE component, ChlF is sensitive to environment that a different set of genes influences the studied traits differently and that responses of genotypes with respect to the studied traits may not be consistent across environments.

Accuracy of GP and PP were evaluated using 3-fold cross-validation with the final model performance computed as the average over 20 replications. For all statistical models, except for GBLUP, we evaluated the performance of PP by using ChlF data instead of the SNPs for each hybrid.

### Comparison of trait predictability based on GP and PP models for 3 traits and identification of the best-performing H3W coffee family

**Fig. 1.**
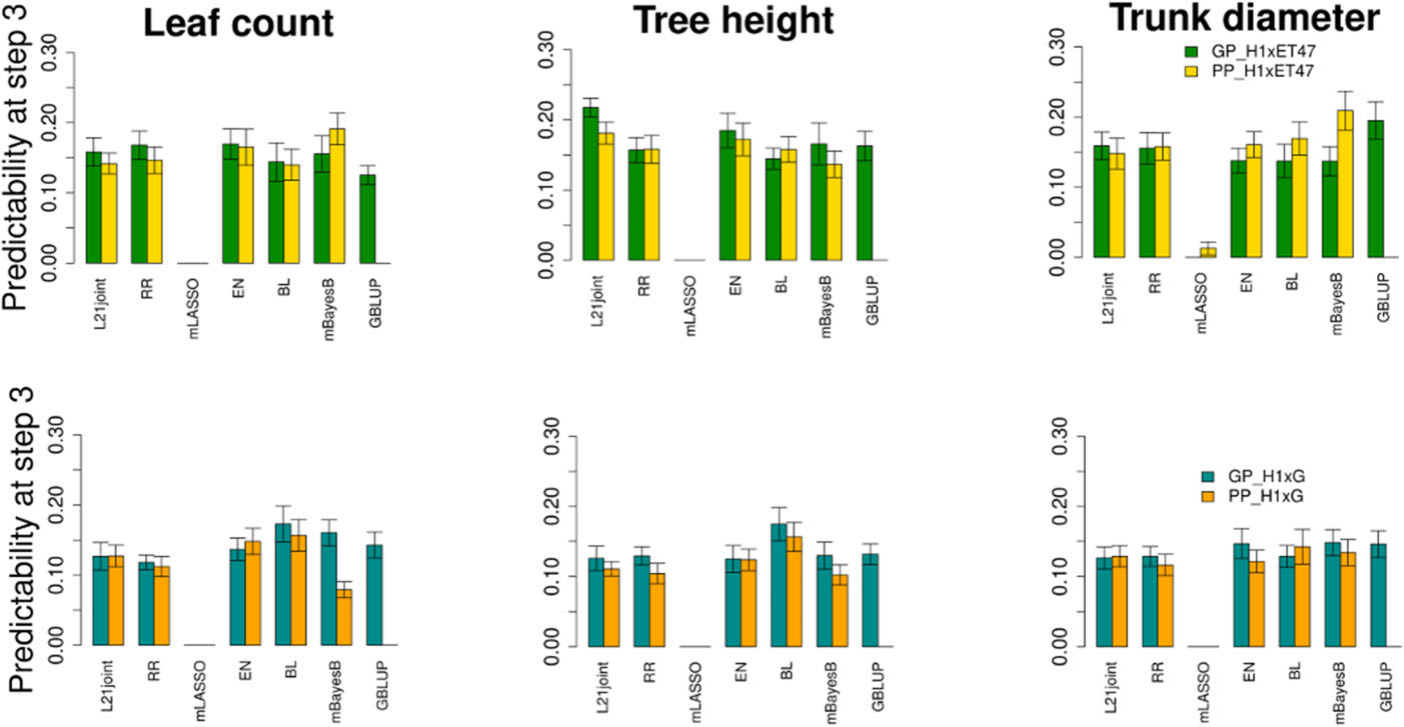
Predictability of traits in H3W coffee families based on GP and PP models. We used the following models: L21-joint, RR, mLASSO, EN, BL, mBayesB, and GBLUP to predict LC (left), TH (middle), and TD (right). This is setting S1 with traits and phenomic data obtained by concatenating the respective measurements over all conditions after the acclimation. The predictability is computed as the average Pearson correlation coefficient between observed and predicted values for the 9 traits (i.e. 3 traits for each treatment) in the validation set, based on 20 repetitions of 3-fold cross-validation. Two H3W coffee populations were considered for the comparative analysis: H1xET47 and H1xG, where Centroamericano (H1) is an F1 hybrid cultivated clonally and results from a cross between T.05296 and Rume Sudan, and Geisha 3 (G) and ET47 (the mother plant) are 2 Ethiopian landrace varieties. The average accuracy obtained from repeated cross-validations are reported as the height of the bars, and standard errors are included.

To assess the predictability of 3 growth-related traits, namely, LC, TH, and TD, we consider setting S1 to built and compared 7 models [i.e. L21-joint, RR, multi-response LASSO (mLASSO), EN, BL, mBayesB, and GBLUP] based on SNPs for GP and ChlF data in 3 treatments (see Table 1) for PP for the H1xET47 and H1xG families. Our findings show that under all treatment conditions, GP and PP models from the H1xET47 family achieve the highest predictability for all traits of interest (Fig. 1 and Supplementary Fig. 1). Furthermore, with Hotelling’s $T^{2}$ test (Hotelling 1992) indicating significant statistical difference (P-value=0.0002876) between the mean performance of the 2 population, we can conclude that the traits for H1xET47 hybrids can be predicted better than those from H1xG. Moreover, for all hybrids and traits, the highest predictability was seen at treatment 3 and the lowest was always exhibited by mLASSO.

Within population and for all treatment conditions, a clear decision regarding the systematic outperformance of GP or PP could not be made because the highest predictability for the traits of interest was achieved in at least 1 combination of population and treatment by each approach. As shown by Supplementary Tables 4 and 5 where the maximum genetic correlation (Galic *et al.* 2019) between the growth-related traits and ChlF measurements are respectively 0.35 and 0.38 for H1xET47 and H1xG, one may favor GP because ChlF parameters seem to have small heritability ($0\leq H^{2}\leq 0.24$, Supplementary Tables 2 and 3). However, ChlF can be used as a valuable predictor because increased electron transport efficiency leads to better carbon partitioning (Ni *et al.* 2009; Shen *et al.* 2015; Ko *et al.* 2016; Toniutti *et al.* 2019). Furthermore, the chlorophyll content measured on plants cultivated either in phytotron or in field (i.e. full sun and shade) always being higher in hybrids compared to line varieties together with the strong relationship between ChlF and the expression of genes related to the photosynthetic electron transport chain (Toniutti *et al.* 2019) allowed to define PI, the chlorophyll content, and the oxidative stress level as indicators of productivity and plant health. This indicates that ChlF is a good proxi for hybrid vigor. This vigor is translated in Arabica by a faster development of the seedling, which can be measured by the diameter at the collar, the size of the plant, or its number of leaves. We then conclude that PP models compete with the GP counterpart when predicting vigor in H3W coffee at an early developmental stage.

### Comparative analysis of GP and PP under AFS

**Fig. 2.**
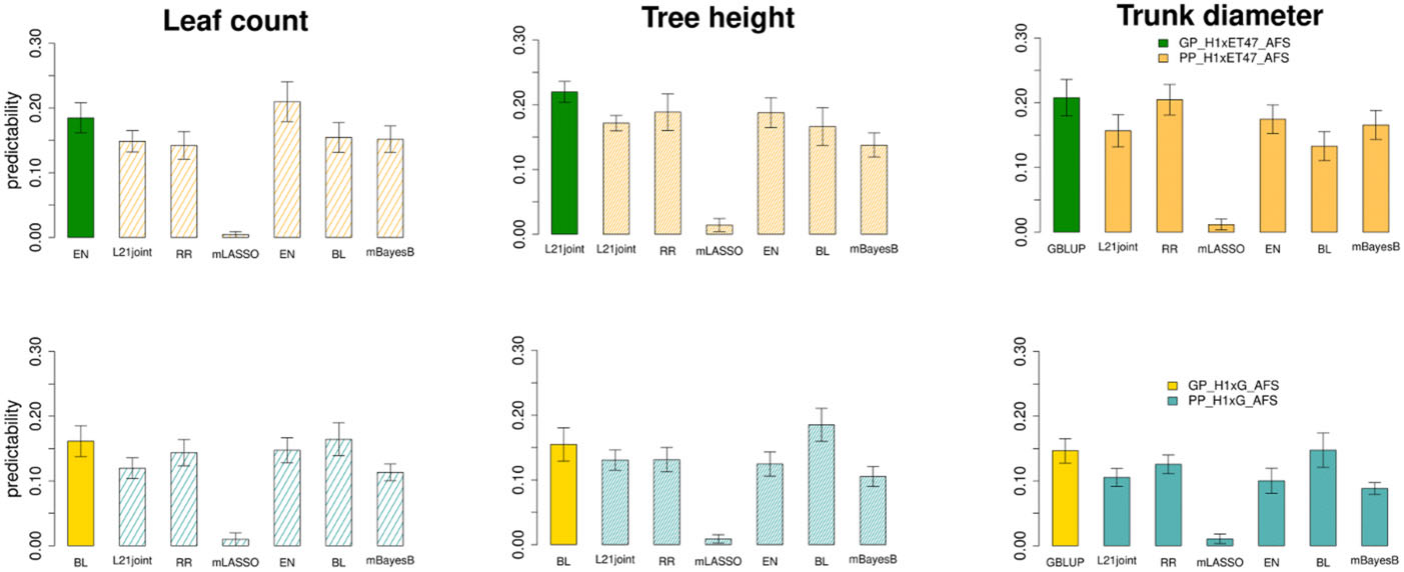
Comparison between GP and PP models under AFS conditions. We used L21-joint, RR, mLASSO, EN, BL, and mBayesB for PP and the best-performing GP model for each H3W coffee plant and trait. For the selected traits, BL and GBLUP are the best-performing GP models for H1xG, while EN, L21-joint, and GLUP are the best GP models for H1xET47. The predictability is computed as the average Pearson correlation coefficient between observed and predicted trait values in the validation set based on 20 replicates of 3-fold cross-validation. The comparative analysis is concerned with setting S2 where the best-performing genomic prediction models for H1xET47 and H1xG populations (i.e. GP-H1xET47 and GP-H1xG) using their respective SNP data, are contrasted with phenomic predictions of the same hybrid families (PP-H1xET47 and PP-H1xG) under established AFS. Models were evaluated after treatment 3 (Table 1) with phenotypic and phenomic data following setting S2. The average accuracy obtained from repeated cross-validations are presented as the height of the bars along with their corresponding standard errors.

Performance comparison of GP and PP was conducted with L21-joint, RR, mLASSO, EN, BL, and mBayesB to predict each of the 3 growth-related traits under 50% shade net equivalent to established AFS (i.e. setting S2). As depicted in Fig. 2, our results show that under AFS, for H1xG hybrids and the corresponding ChlF data, the best-performing PP and GP model outperformed each other on 1 count out of 3 and achieved similar predictability for the remaining trait. With respect to the H1xET47 population, a similar pattern could be observed, whereby L21-joint and EN are the GP and PP models with the highest predictability for TH and LC, respectively.

Because a single genotyping experiment suffices to determine the predictors used in GP models, while multiple phenomic data collection at different stages of growth may be needed to obtain reliable predictability of PP models, one may argue that GP should be favored. However, SNPs in polyploid and heterozygote species, like *C.arabica*, are more difficult to determine, while endophenotypes (e.g. ChlF) can be easily measured. Since PP relied only on 18 predictors for each treatment condition and GP on 16,950 SNPs, and because genotyping (i.e. about 220 €/sample) in this case is more expensive than phenomic data collection (i.e. 5,300 €for all samples including fluorimeter purchase), our results indicate that PP can be a competitive approach in predicting growth-related traits in coffee while requiring some efforts to obtain the endophenotypes.

### Predictability of traits based on PP models and the effect of including more predictors

To see if including ChlF measurements from all conditions impacts the performances of PP models for the 2 H3W families, we considered changes with respect to established AFS conditions. To this end, plants were moved from shade and exposed under full sun, the altitude level was increased by 700 m, and temperature decreased to 20°C, as described in Table 1. To account for these changes, we make use of the second aim of setting S2 with phenomic measurements concatenated over all treatments except the acclimation, and the phenomic predictive ability on the traits of interest evaluated. With respect to the best-performing statistical model, our findings show an increased PP accuracy with the augmented fluorescence data model for both H1xET47 and H1xG families on 2 out of 3 traits (i.e. LC and TD, Supplementary Fig. 2). Moreover, when considering only H1xET47, a clear pattern of improved predictability for the augmented model could be observed for all traits with the multi-trait models (i.e. mBayesB and L21-joint). The inclusion of additional predictors from different environmental conditions exhibiting a change on PP performances and especially for multi-trait models could suggest that ChlF measurements over different growth periods could be helpful in boosting the model performance.

### A comparative analysis of GP and PP models using condition-ahead prediction

Our interest with condition-ahead prediction (i.e. setting S3) is to further compare the performances of GP and PP models based on their abilities to predict the next environmental condition while being trained on the previous one (e.g. use treatment 2 as training data to predict the corresponding trait values in treatment 3). With phenomic and trait data from H1xET47 and H1xG at the targeted training treatment, we start by estimating GP and PP models’ parameters before using them to predict the first 20 lines in the corresponding test data. Because SNPs are recorded only once and for a fair comparison between GP and PP, we trained the models without the first 20 samples, such that they can be used as unseen data in the prediction phase. Our results show that, with H1xET47 and when the models were trained with data from acclimation to predict traits under established AFS, GP outperformed PP on 2 counts (i.e. for TH and TD) out of 3 as quantified by the correlation coefficient between measured and predicted traits values and reported in Table 2. Compared to the performance with GP when training the model with data from established AFS to predict traits under higher altitude, LC and TD were better predicted using PP. With the family H1xG, for all traits and under all training and predictions settings, PP and GP achieved in most cases comparable predictability with slight improvement observed for PP on some occasions. Because the highest predictabilities were mostly observed with PP models for both crosses, we conclude that PP models exhibit better performance. This is likely due to the fact that different ChlF data are recorded in each environmental conditions and accounted for in the training process of PP, while the same SNPs are constantly used across environments for GP. The highest accuracies often occurring when GP and PP models for both families were trained under established AFS to predict traits in treatment 4 could also suggest that models reach their best-training abilities under shade. Under setting S4 and since the 2 families have 1 parent (i.e. H1) in common, we next considered comparing the predictabilities of GP and PP models trained with data from H1xET47 and evaluated on traits from H1xG and vice versa. With traits and fluorescence data constructed in setting S1, our results in Table 3 show that PP models exhibit higher predictability than GP model of the considered traits when transferred from H1xET47 to H1xG, but not vice versa. This findings shows that while PP models may be a suitable alternative to GP, they have similar problems to the transferability of models on unseen populations.

**Table 2.** Comparison between GP and PP models based on condition-ahead predictive abilities.

H1xET47												
GP of treatment 3 using treatment 2							PP of treatment 3 using treatment 2					
	BL	EN	GBLUP	L21-joint	mBayesB	RR		BL	EN	L21-joint	mBayesB	RR
LC	0.276	0.164	0.086	0.06	0.032	**0.34**	LC	0.429	**0.539**	0.376	0.387	0.315
TH	0.055	0.324	**0.419**	0.307	0.063	0.186	TH	0.297	0.076	0.303	**0.392**	0.083
TD	0.016	0.049	0.079	0.164	**0.516**	0.115	TD	0.273	**0.491**	0.205	0.104	0.314

**GP of treatment 4 using treatment 3**							**PP of treatment 4 using treatment 3**					
	
	**BL**	**EN**	**GBLUP**	**L21-joint**	**mBayesB**	**RR**		**BL**	**EN**	**L21-joint**	**mBayesB**	**RR**

LC	**0.191**	0.124	0.012	0.111	0.153	0.068	LC	**0.224**	0.167	0.111	0.012	0.163
TH	0.182	0.168	**0.493**	0.483	0.188	0.271	TH	0.146	0.107	**0.418**	0.317	0.051
TD	0.202	**0.338**	0.028	0.23	0.082	0.095	TD	0.36	**0.365**	0.165	0.042	0.056

**GP of treatment 4 using treatment 2**							**PP of treatment 4 using treatment 2**					
	
	**BL**	**EN**	**GBLUP**	**L21-joint**	**mBayesB**	**RR**		**BL**	**EN**	**L21-joint**	**mBayesB**	**RR**

LC	0.279	0.122	0.106	0.173	0.173	**0.484**	LC	0.0187	**0.495**	0.023	0.083	0.084
TH	0.204	0.168	0.427	**0.482**	0.036	0.052	TH	0.359	0.131	**0.496**	0.382	0.223
TD	0.004	0.154	0.047	**0.239**	0.061	0.13	TD	0.069	0.138	0.0425	**0.369**	0.287

**H1xG**												

**GP of treatment 3 using treatment 2**							**PP of treatment 3 using treatment 2**					
	
	**BL**	**EN**	**GBLUP**	**L21-joint**	**mBayesB**	**RR**		**BL**	**EN**	**L21-joint**	**mBayesB**	**RR**

LC	**0.197**	0.081	0.026	0.218	0.191	0.142	LC	**0.213**	NA	0.069	0.098	0.052
TH	0.043	0.009	**0.117**	0.112	0.065	0.022	TH	0.105	0.09	0.07	0.194	**0.289**
TD	**0.314**	0.125	0.116	0.069	0.025	0.372	TD	0.094	0.072	**0.316**	0.024	0.023

**GP of treatment 4 using treatment 3**							**PP of treatment 4 using treatment 3**					
	
	**BL**	**EN**	**GBLUP**	**L21-joint**	**mBayesB**	**RR**		**BL**	**EN**	**L21-joint**	**mBayesB**	**RR**

LC	**0.14**	0.037	0.133	0.121	0.072	0.005	LC	0.294	0.186	0.332	0.181	**0.369**
TH	0.08	0.076	0.053	0.188	0.094	**0.355**	TH	**0.508**	0.37	0.03	0.13	0.136
TD	0.012	0.23	0.059	**0.35**	0.132	0.155	TD	0.207	0.01	0.151	0.097	**0.359**

**GP of treatment 4 using treatment 2**							**PP of treatment 4 using treatment 2**					
	
	**BL**	**EN**	**GBLUP**	**L21-joint**	**mBayesB**	**RR**		**BL**	**EN**	**L21-joint**	**mBayesB**	**RR**

LC	0.334	0.103	0.136	0.167	0.096	**0.383**	LC	0.279	NA	0.191	**0.389**	0.386
TH	0.027	0.11	0.068	0.081	0.063	**0.361**	TH	0.434	0.381	0.381	**0.582**	0.305
TD	0.015	0.175	0.036	0.149	0.19	**0.288**	TD	0.018	0.087	0.231	**0.2964**	0.149

We used L21-joint, RR, mLASSO, EN, BL, and mBayesB. The performance is computed as the correlation coefficient between measured and predicted LC, TH, and TD, for H1xET47 (i.e. top panel) and H1xG (i.e. bottom panel). This is setting S3, where models are trained on the current environmental condition to predict the next one. Numbers in bold represent the best performance and mLasso is not represented because all the corresponding standard deviations were zero.

**Table 3.** Comparison between GP and PP models based on between-family predictive abilities.

GP of H1xG using H1xET47								PP of H1xG using H1xET47					
	BL	EN	GBLUP	L21-joint	mBayesB	mLasso	RR	BL	EN	L21-joint	mBayesB	mLasso	RR
LC2	0.038	0.13	0.056	0.038	0.114	0.03	**0.308**	0.099	0.137	0.009	0.102	NA	0.02
TH2	0.002	0.095	0.022	0.061	0.168	0.072	0.103	**0.305**	0.123	0.236	0.294	NA	0.094
TD2	0.025	0.07	0.078	0.007	0.079	0.168	0.042	0.111	0.273	0.01	0.225	NA	**0.359**
LC3	0.1	0.118	0.046	0.003	0.052	0.194	0.222	0.027	0.164	0.128	0.177	NA	**0.223**
TH3	0.066	0.203	0.035	0.083	0.127	0.039	0.073	**0.345**	0.153	0.168	0.322	NA	0.032
TD3	0.078	**0.209**	0.066	0.168	0.076	0.114	0.079	0.14	0.018	0.138	0.096	NA	0.058
LC4	0.025	0.095	0.064	0.037	0.143	0.108	0.138	0.144	0.087	0.096	0.143	NA	**0.167**
TH4	0.009	0.09	0.068	0.128	0.018	0.015	0.224	0.252	0.084	0.277	**0.279**	NA	0.051
TD4	0.069	0.074	0.154	0.027	0.114	0.139	0.051	0.211	0.096	**0.214**	0.163	NA	0.039

**GP of H1xET47 using H1xG**								**PP of H1xET47 using H1xG**					
	
	**BL**	**EN**	**GBLUP**	**L21-joint**	**mBayesB**	**mLasso**	**RR**	**BL**	**EN**	**L21-joint**	**mBayesB**	**mLasso**	**RR**

LC2	0.201	0.025	**0.343**	0.072	0.29	NA	0.008	0.118	0.066	0.081	0.018	NA	0.098
TH2	0.143	0.169	0.078	0.04	0.006	NA	**0.349**	0.008	0.002	0.116	0.047	NA	0.053
TD2	0.009	0.196	**0.204**	0.111	0.127	NA	0.161	0	0.037	0.054	0.007	NA	0.052
LC3	0.07	**0.197**	0.052	0.157	0.106	NA	0.025	0.005	0.11	0.018	0.021	NA	0.144
TH3	0.016	**0.214**	0.077	0.122	0.029	NA	0.194	**0.129**	0.002	0.126	0.04	NA	0.082
TD3	0.038	0.021	0.142	0.099	0.117	NA	0.093	0.086	0.068	0.067	0.151	NA	**0.326**
LC4	**0.213**	0.101	0.036	0.002	0.142	NA	0.036	0.096	0.103	0.033	0.064	NA	0.058
TH4	0.004	0.116	**0.214**	0.118	0.002	NA	**0.214**	0.096	0.09	0.048	0.003	NA	0.049
TD4	**0.345**	0.186	0.194	0.125	0.294	NA	0.244	0.133	0.203	0.178	0.111	NA	0.08

We used L21-joint, RR, mLASSO, EN, BL, and mBayesB. The performance is computed as the correlation coefficient between measured and predicted LC, TH, and TD at each treatment condition and for H1xET47 (i.e. top panel) and H1xG (i.e. bottom panel). This is setting S4, where models are trained with data from one family to predict traits of the other one, with traits and phenomic data constructed by concatenating the respective measurements over all treatment conditions after the acclimation period. Numbers in bold represent the best performance and NA is used to denote that the corresponding standard deviation was zero.

### Model performances based on the selection ability of the best- and worst-performing lines

To further assess the performance of GP and PP models on each H3W population, by ranking the genotypes based on the measured and predicted values of each trait. The 20 best- and worst-performing lines for each category were then retained and used to compute the proportion of the best- and worst-performing lines that were correctly predicted as the best and worst performing, respectively. Our findings in Table 4 identified on 2 counts EN as the best-performing GP model for H1xG family, whereas L21-joint outperformed the contenders when LC and TH were the traits of interest using H1xET47 population. Decision regarding the best statistical model with respect to H3W family and condition could not be made because each model was ranked first at least once, for a specific trait. However, one can observe that the highest performances were attained under PP with trees from H1xET47 family (Table 4).

**Table 4.** Selection performance of L21-joint, RR, mLASSO, EN, BL, and mBayesB.

Selected proportion of best-performing lines														
	RR	Mlasso	EN	GBLUP	BL	mBayesB	L21-joint	RR	Mlasso	EN	GBLUP	BL	mBayesB	L21-joint
**(A1): GP_H1xG**								**(A2): GP_H1xET47**						

LC	15	**20**	5	**20**	10	5	**20**	15	20	25	25	25	35	**30**
TH	15	10	**20**	5	15	15	15	30	20	20	20	30	30	**35**
TD	15	10	**25**	20	15	15	20	10	10	15	**25**	10	20	**25**

**(B1): PP_H1xG**								**(B2): PP_H1xET47**						

LC	15	10	10	xx	**20**	**20**	**20**	25	5	25	xx	30	25	30
TH	0	10	15	xx	15	5	10	20	15	**35**	xx	30	25	30
TD	5	10	**35**	xx	15	15	25	**30**	25	25	xx	25	**30**	**30**

The performance is computed as the proportion of correctly selected best-performing lines with respect to LC, TH, and TD. For populations H1xG (i.e. left panel) and H1xET47 (i.e. right panel), the assessment is conducted for genomic and phenomic prediction models accounting for environmental conditions. Numbers in bold represent the best performance and we write xx to express that the corresponding statistical approach was not used for phenomic prediction.

Regarding the selection ability on the worst-performing lines (Supplementary Table 6), similar conclusions can be reached, whereby for GP models on H1xG family, GBLUP outperformed the contenders when predicting LC and TH and L21-joint was the best-performing model for the same traits with H1xET47 family. In addition, we still observed at the population level that the highest ability for negative selection (i.e. proportion of the worst-performing lines predicted as worst performing) was achieved with PP on H1xET47 family.

## Conclusion

Our comparative analyses provided a comprehensive investigation of the differences in the performance of GP and PP models for 3 growth-related traits from 2 H3W coffee families exposed to a succession of treatments. The PP models are based on measurements of ChlF after the exposure to each environmental condition. The comparative analyses contrasted 7 different statistical models that differ with respect to whether they are aimed at predicting single trait or MTs. In the 3 considered settings for the comparison of PP and GP models within and between H3W coffee families, we showed that, although ChlF parameters in both H1xET47 and H1xG seem to have small heritabilities ($0\leq H^{2}\leq 0.24$), PP tends to outperform GP models and ChlF can be used as a suitable alternative to genomic markers when predicting plant vigor. Interestingly, however, in the fourth scenario that tests the transferability of the models between the families, we showed that PP suffers the same issues as GP models, and here, the consideration of more phenomic data (e.g. NIRS) may improve the performance.

In Toniutti *et al.* (2017) and Gamboa-Becerra *et al.* (2021), it has been demonstrated that parameters related to photosystem II and photosynthetic electron transport chain components are powerful indicators of the physiological status of the coffee plants and predict infection intensity, respectively, of *Hemileia vastatrix* and *Fusarium* isolates, in combination with different kinds of abiotic stress. These works highlight the relevance of ChlF as an early and high-throughput phenotyping tool for plant stress. Although the mechanisms underlying heterosis remain largely unknown, several recent studies have shown that hybrid vigor is due, at least in part, to a deregulation of certain central genes of the circadian cycle. Ni *et al.* (2009) showed that, in *Arabidopsis* hybrids and allopolyploids, increased photosynthetic and metabolic activities are linked to altered expression of 2 central genes of the circadian clock. The authors demonstrated that an epigenetic deregulation of circadian clock regulators, which control many genes and are involved in many biological processes, resulted in an increase in chlorophyll content and starch biosynthesis leading to growth vigor and increased biomass (Miller *et al.* 2012). Monocots like maize and rice produced similar results (Song *et al.* 2010; Ko *et al.* 2016). For example, Shen *et al.* (2015) showed that deregulation of 3 circadian clock genes and consequently the downstream genes involved in the chlorophyll and starch metabolic pathways could also be related to heterosis. Toniutti *et al.* (2019) demonstrated a similar relationship between circadian cycle dysregulation and carbon metabolism in coffee tree and established the relationship between the increased photosynthetic electron transport efficiency and the clone’s better performance. ChlF measurement is a good indicator of the coffee tree’s physiological status for the breeder and is an excellent proxy for photosynthesis in coffee, making it a tool of choice for assessing the vigor of a genotype, which the present study tends to prove.

## Data availability

We implemented all statistical models using R programming language; the codes and all data sets used in the current study are freely available from https://github.com/alainmbebi/GP-PP.

Supplemental material is available at *G3* online.

## Supplementary Material

jkac170_Supplementary_Data_File_S1

jkac170_Supplementary_Data_File_S2
