# Supplementary material for: A comparative analysis of genomic and phenomic predictions of growth-related traits in 3-way coffee hybrids
Source: G3 (Bethesda). 2022 Jul 6;12(9):jkac170. doi: 10.1093/g3journal/jkac170 (PMC9434219; doi:10.1093/g3journal/jkac170)
Supplement: jkac170_Supplementary_Data_File_S1 [file jkac170_supplementary_data_file_s1.pdf]

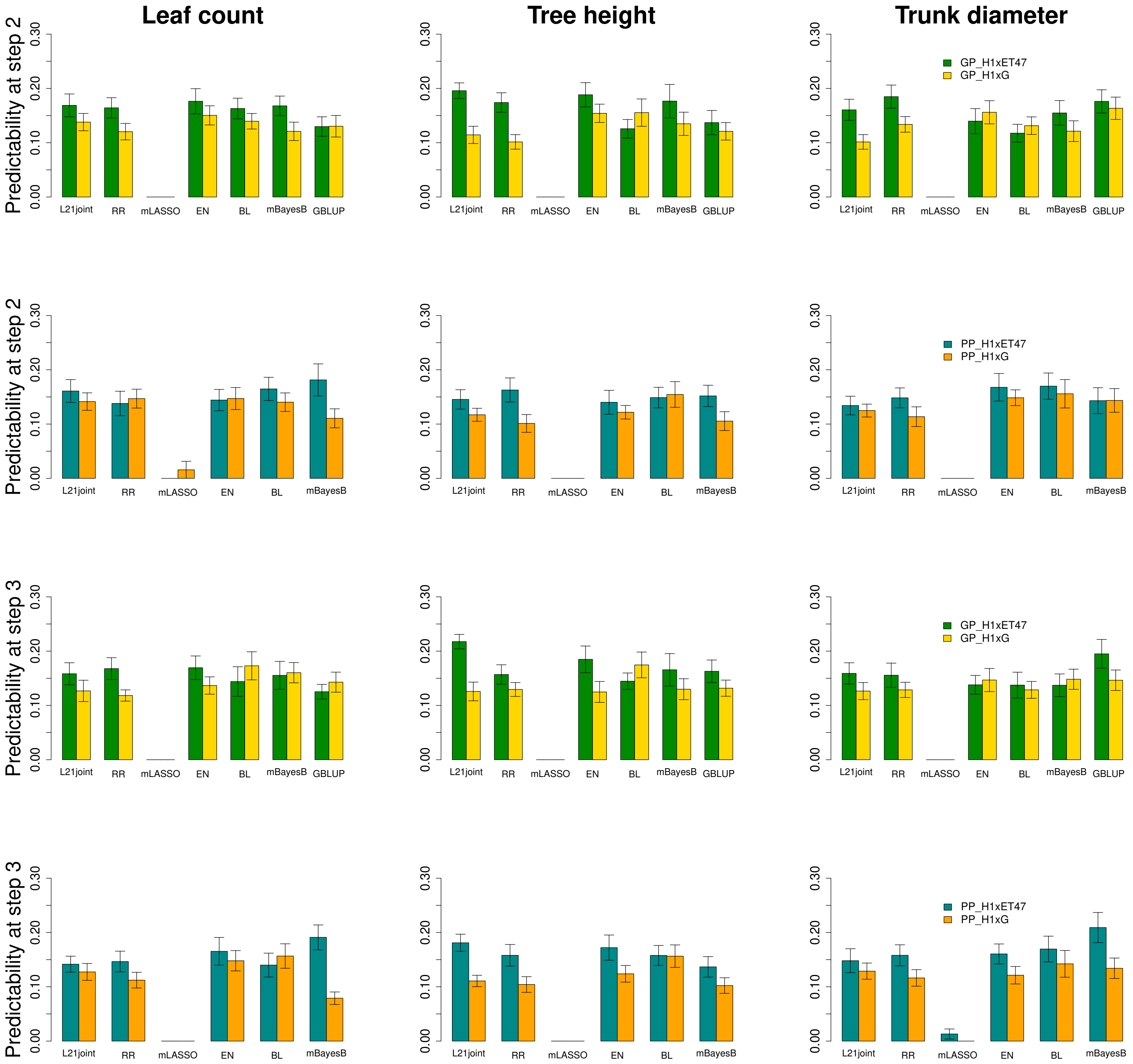

Figure F1: Predictability of traits in H3W coffee families based on GP and PP models. We used the following models: L21joint, ridge regression (RR), multiple LASSO (mLASSO), elastic-net (EN), Bayesian LASSO (BL), multiple-trait BayesB (mBayesB) and GBLUP to predict leaf count (left), tree height (middle) and trunk diameter (right). This is Setting S1 with traits and phenomic data obtained by concatenating the respective measurements over all stress conditions after the acclimation. The predictability is computed as the average Pearson correlation coefficient between observed and predicted values for the nine traits (i.e. three traits for each treatment) in the validation set, based on 20 repetitions of 3-fold cross-validation. Two H3W coffee populations were considered for the comparative analysis: H1xET47 and H1xG, where, Centroamericano (H1) is an F1 hybrid cultivated clonally and results from a cross between T.05296 and Rume Sudan, and Geisha 3 (G) and ET47 (the mother plant) are two Ethiopian landrace varieties. The average accuracy obtained from repeated cross-validations are reported as the height of the bars, and standard errors are included.

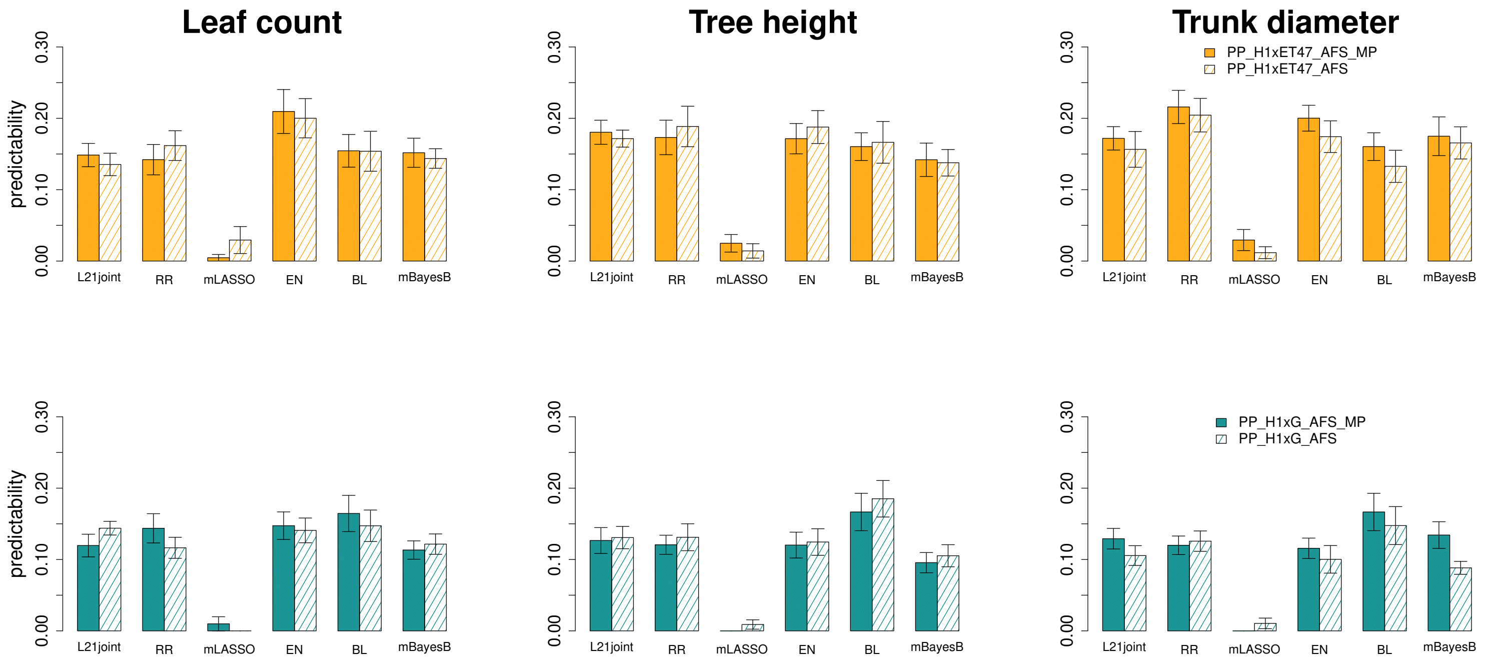

Figure F2: Predictability of traits based on PP models and the effect of including more predictors. We used L21-joint, ridge regression (RR), multiple LASSO (mLASSO), elastic-net (EN), Bayesian LASSO (BL) and multiple-trait BayesB (mBayesB) for each H3W coffee plant and trait. The performance is computed as the average Pearson correlation coefficient between observed and predicted trait values in the validation set based on 20 replicates of 3-fold cross-validation. The comparative analysis contrast the predictability of PP models for H1xET47 and H1xG populations under AFS conditions (i.e. PP-H1xET47-AFS and PP-H1xG-AFS) with the scenario where additional fluorescence measurements from treatment 3 and 4 are included in the model (i.e. PP-H1xET47-AFS-MP and PP-H1xG-AFS-MP). Traits in the two cases were the same and only the set of predictors varied (i.e. 18 for AFS and 54 for AFS-MP). The average accuracy obtained from repeated cross-validations are presented as the height of the bars along with their corresponding standard errors.

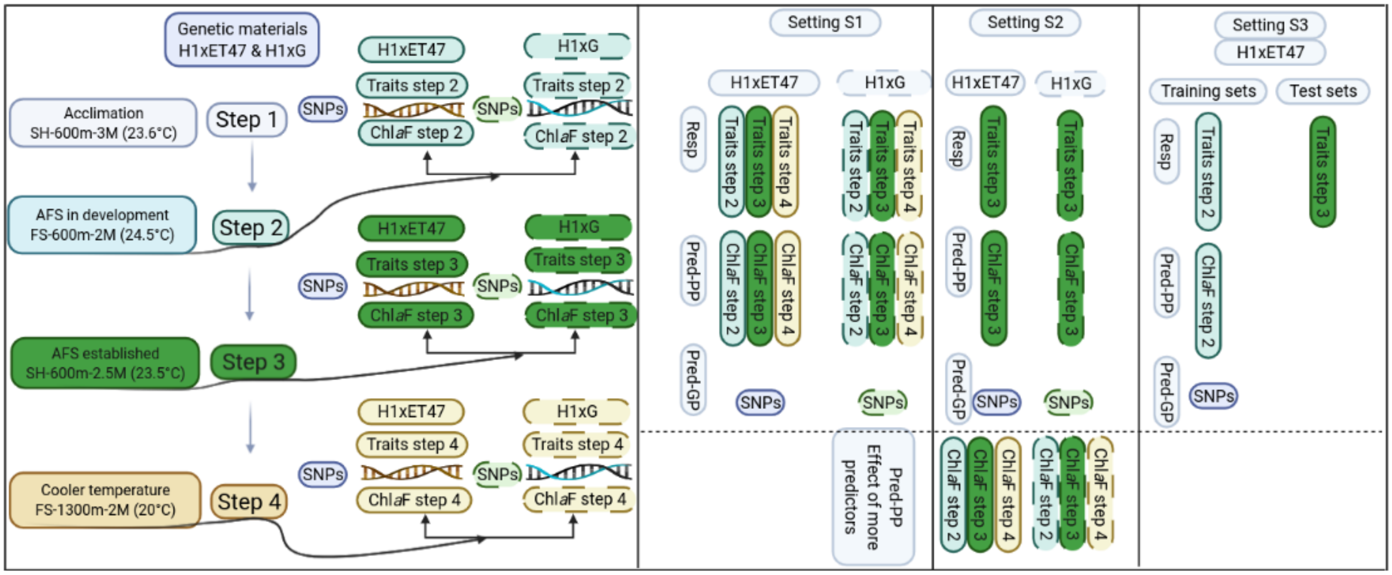

Figure F3: Graphical representation of data construction for each setting used in the comparative analysis. Treatment/step 1 correspond to the acclimation period where plants were grown for 3 months under shade, at 600m of altitude and temperature 23.6°C (SH-600m-3M). Under treatment/step 2, open field (i.e. AFS in development), plants were exposed to full sun for 2 months at altitude level 600m and ambient temperature 24.5°C (FS-600m-2M). During treatment/step 3 that lasted 2.5 months, the altitude level was unchanged (i.e. 600m), but temperature decreased to 23.5°Cand plants moved back under shade (SH-600m-2.5M). Finally, at treatment/step 4 and during 2 months, plants experienced cooler temperature (i.e. 20°C) and higher altitude (i.e. 1300m) (FS-1300m-2M). Chl*a*F, Pred-PP, Pred-GP and Resp, denote respectively chlorophyll *a* fluorescence measurements, predictors used in PP, predictors used in GP and the response variables. In Setting S3, we only show a representative case for H1xET47 and the remaining cases can be constructed similarly. Setting S4 is not shown because its use similar data as in Setting S1.
