# Supplementary material for: A comparative analysis of genomic and phenomic predictions of growth-related traits in 3-way coffee hybrids
Source: G3 (Bethesda). 2022 Jul 6;12(9):jkac170. doi: 10.1093/g3journal/jkac170 (PMC9434219; doi:10.1093/g3journal/jkac170)
Supplement: jkac170_Supplementary_Data_File_S2 [file jkac170_supplementary_data_file_s2.pdf]

Table T1: Energy fluxes and Chlorophyll *a* fluorescence variables. PSI, PSII, RC, CS, and Q_A stand respectively for photosystem I, photosystem II, total number of active PSII reaction centers in the measured area, the cross section of PSII, and the first plastoquinone electron acceptor of PSII. As for energy fluxes parameters, we have: The photon flux absorbed by the antenna of PSII units (ABS). The part of ABS trapped by the active PSII units that leads to Q_A reduction (TR). The part of ABS dissipated in PSII antenna in processes other than trapping (DI). The energy flux associated with the electron transport from Q_A- to the intersystem electron acceptors (ET) and the energy flux associated with the electron transport from Q_A- to the final electron acceptors of PSI (RE). Note that, a parameter followed by subscripts P/M or O, means its corresponding peak/maximal and minimal values respectively (e.g. the maximal fluorescence F_M).

Technical fluorescence parameters	Meaning
$F_V = F_M - F_o$	Maximum variable fluorescence
$V_t = (F_t - F_o)/F_V$	Relative variable fluorescence
$M_o = (\Delta V/\Delta t)0 \approx 4(F_{0.3ms} - F_{0.05ms})/F_V$	Initial slope (in ms^{-1}) of the O-J fluorescence rise
$Sm = Area/F_V$	Normalized area between the OJIP curve and the line $F = F_M$, which is a proxy of the number of electron carriers per electron transport chain
**Efficiencies and quantum yields**	
$ET_o/TR_o = \Psi E_o = 1 - V_J$	Efficiency with which a PSII trapped electron is transferred from Q_A- to PQ
$RE_o/TR_o = \Psi R_o = 1 - V_I$	Efficiency with which a PSII trapped electron is transferred to final PSI acceptors
$RE_o/ET_o = \delta R_o = \Psi R_o/\Psi E_o$	Efficiency with which an electron from PQH_2 is transferred to final PSI acceptors
$TR_o/ABS = \varphi P_o = F_V/F_M$	Maximum quantum yield of primary PSII photochemistry
$ET_o/ABS = \varphi E_o = \varphi P_o \times \Psi E_o$	Quantum yield of electron transport from Q_A- to PQ
$RE_o/ABS = \varphi R_o = \varphi P_o \times \Psi R_o$	Quantum yield of electron transport from Q_A- to final PSI acceptors
$DI_o/ABS = 1 - TR_o/ABS$	Quantum yield of energy dissipation in PSII antenna
**Specific energy fluxes (per active PSII)**	
$ABS/RC = (M_o/V_J)/\varphi P_o$	Apparent antenna size of an active PSII
$TR_o/RC = M_o/V_J$	Maximum trapped exciton flux per active PSII
$ET_o/RC = (M_o/V_J) \times \Psi E_o$	The flux of electrons transferred from Q_A- to PQ per active PSII
$RE_o/RC = (M_o/V_J) \times \Psi R_o$	The flux of electrons transferred from Q_A- to final PSI acceptors per active PSII
$DI_o/RC = ABS/RC - TR_o/RC$	The flux of energy dissipated in processes other than trapping per active PSII
**Phenomenological energy fluxes (per CS)**	
$ABS/CS_M \approx F_o$ and $ABS/CSM \approx F_M$	Absorbed photon flux per excited cross section of PSII
$TR_o/CS = (TR_o/ABS) \times (ABS/CS)$	Maximum trapped exciton flux per cross section of PSII
$ET_o/CS = (ET_o/ABS) \times (ABS/CS)$	The flux of electrons from Q_A- to PQ per cross section of PSII
$RE_o/CS = (RE_o/\mathit{ABS}) \times (ABS/CS)$	The flux of electrons from Q_A- to final PSI acceptors per cross section of PSII
Performance indexes	Performance of PSII and of specific electron transport reactions
$PI_{ABS} = (RC/ABS)\times [\varphi P_o/(1 - \varphi P_o)] \times [\Psi E_o/(1 - \Psi E_o)]$	Performance index on absorption basis
$PI_{ABS,total} = PI_{ABS} \times [\delta R_o/(1 - \delta R_o)]$	Total performance index on absorption basis
$PI_{CS} = PI_{ABS} \times (ABS/CS)$	Performance index on cross section basis
$PI_{CS,total} = PI_{ABS,total} \times (ABS/CS)$	Total performance index on cross section basis
**Driving forces**	**Performance of PSII and of specific electron transport reactions**
$DF_{ABS} = \log(PI_{ABS})$	Driving force on absorption basis
$DF_{ABS,total} = \log(PI_{ABS,total})$	Total driving force on absorption basis
$DF_{CS} = \log(PI_{CS})$	Driving force on cross section basis
$DF_{CS,total} = \log(PI_{CS,total})$	Total driving force on cross section basis

■ **Table T2:** Broad-sense (trait) heritability across multiple environments for H1xET47 family along with partition of variance between genetic (G), environment (E), genetic by environment (G $\times$ E) and residual (ε). The performance is computed for the three target traits (i.e. leaf count (LC), tree height (TH) and trunk diameter (TD)), and for the 18 chlorophyll *a* fluorescence variables (i.e. phenomic). Note that we considered 3 different environments corresponding to treatment conditions 2, 3 and 4. Zero entries correspond to the case where the variance components were very small.

Traits	$\sigma^2_{\mathbf{G}}$	$\sigma^2_{\mathbf{E}}$	$\sigma^2_{\mathbf{GxE}}$	σ^2_{ε}	$\%(\mathbf{G})$	$\%(\mathbf{E})$	$\%(\mathbf{G} \times \mathbf{E})$	$\%(\varepsilon)$	$\mathbf{H}^2$
**LC**	3282.208	3098.041	2438.364	2784.698	0.283	0.267	0.21	0.24	0.404
**TH**	37815.966	20800.707	110.291	6907.634	0.576	0.317	0.002	0.105	0.62
**TD**	5.051	23.192	0.005	3.62	0.159	0.728	0	0.114	0.172
**IBR**	0	1.066	7252.622	1994.633	0	0	0.784	0.216	0
$\mathbf{PI_{total}}$	0.072	0.081	0.769	0.152	0.067	0.075	0.716	0.142	0.157
$\varphi\mathbf{R_o}$	0	0	0	0	0	0.152	0.669	0.179	0
$\Psi\mathbf{E_o}$	0	0	0.001	0.001	0	0.001	0.66	0.339	0
$\varphi\mathbf{E_o}$	0	0	0.002	0	0	0.056	0.732	0.212	0
$\varphi\mathbf{P_o}$	0	0	0	0	0.099	0.127	0.61	0.164	0.204
$\varphi\mathbf{P_o}/(1-\varphi\mathbf{P_o})$	0.021	0.024	0.088	0.033	0.123	0.147	0.53	0.199	0.24
$\delta\mathbf{R_o}/(1-\delta\mathbf{R_o})$	0	0.006	0.019	0.005	0	0.218	0.63	0.151	0
$\Psi\mathbf{E_o}/(1-\Psi\mathbf{E_o})$	0	0.006	0.035	0.009	0	0.114	0.7	0.186	0
$\mathbf{RC/ABS}$	0.001	0.004	0.01	0.004	0.06	0.192	0.539	0.209	0.12
$\mathbf{RE_o/RC}$	0	0	0	0	0.018	0.333	0.313	0.336	0.032
$\mathbf{DI_o/RC}$	0	0.001	0.007	0.001	0.036	0.057	0.789	0.118	0.091
$\mathbf{ET_o/RC}$	0	0.002	0.002	0.001	0.073	0.39	0.325	0.213	0.114
$\mathbf{TR_o/RC}$	0.001	0.005	0.02	0.006	0.036	0.143	0.63	0.191	0.08
$\mathbf{ABS/RC}$	0.003	0.008	0.051	0.011	0.038	0.106	0.704	0.152	0.088
$\mathbf{F_o}$	71.682	24657.971	277.412	763.856	0.003	0.957	0.011	0.03	0.003
$\mathbf{F_M}$	740.746	506496.856	1.289	25585.391	0.001	0.951	0	0.048	0.001
$\mathbf{F_V/F_M}$	0	0	0	0	0.098	0.127	0.611	0.163	0.203

Table T3: Broad-sense (trait) heritability across multiple environments for H1xG family along with partition of variance between genetic (G), environment (E), genetic by environment (G × E) and residual (ε). The performance is computed for the three target traits (i.e. leaf count (LC), tree height (TH) and trunk diameter (TD)), and for the 18 chlorophyll *a* fluorescence variables (i.e. phenomic). Note that we considered 3 different environments corresponding to treatment conditions 2, 3 and 4. Zero entries correspond to the case where the variance components were very small.

Traits	$\sigma^2_{\mathbf{G}}$	$\sigma^2_{\mathbf{E}}$	$\sigma^2_{\mathbf{GxE}}$	σ^2_{ε}	$\%(\mathbf{G})$	$\%(\mathbf{E})$	$\%(\mathbf{G}\times\mathbf{E})$	$\%(\varepsilon)$	$\mathbf{H}^2$
**LC**	5999.467	3463.762	285.641	5984.008	0.381	0.22	0.018	0.38	0.519
**TH**	62358.85	15852.885	694.241	4830.355	0.745	0.189	0.008	0.058	0.779
**TD**	7.326	30.681	0	4.152	0.174	0.728	0	0.098	0.186
**IBR**	0	0	0	12182.381	0	0	0	1	0
$\mathbf{PI_{total}}$	0.229	0.244	1.748	0.446	0.086	0.091	0.655	0.167	0.19
$\varphi\mathbf{R_o}$	0	0	0	0	0.006	0.022	0.732	0.24	0.018
$\Psi\mathbf{E_o}$	0	0	0.002	0	0	0.101	0.702	0.198	0
$\varphi\mathbf{E_o}$	0	0.001	0.002	0.001	0	0.194	0.62	0.186	0
$\varphi\mathbf{P_o}$	0	0	0.001	0	0.026	0.032	0.715	0.227	0.07
$\varphi\mathbf{P_o}/(1-\varphi\mathbf{P_o})$	0.007	0.003	0.123	0.04	0.041	0.02	0.708	0.231	0.11
$\delta\mathbf{R_o}/(1-\delta\mathbf{R_o})$	0.001	0.001	0.009	0.003	0.083	0.052	0.65	0.216	0.195
$\Psi\mathbf{E_o}/(1-\Psi\mathbf{E_o})$	0	0.035	0.041	0.021	0	0.362	0.425	0.213	0
**RC/ABS**	0	0.006	0.01	0.004	0	0.282	0.529	0.189	0
$\mathbf{RE_o/RC}$	0	0	0	0	0.116	0.284	0.395	0.205	0.193
$\mathbf{DI_o/RC}$	0	0	0.03	0.005	0	0	0.854	0.146	0
$\mathbf{ET_o/RC}$	0	0.001	0.001	0.001	0.02	0.441	0.262	0.277	0.031
$\mathbf{TR_o/RC}$	0	0.006	0.011	0.004	0	0.269	0.537	0.194	0
**ABS/RC**	0	0.005	0.062	0.013	0	0.062	0.78	0.158	0
$\mathbf{F_o}$	254.685	37148.567	0	2273.711	0.006	0.936	0	0.057	0.007
$\mathbf{F_M}$	4033.968	824383.222	538.016	27614.935	0.005	0.962	0.001	0.032	0.005
$\mathbf{F_V/F_M}$	0	0	0.001	0	0.026	0.032	0.708	0.233	0.071

Table T4: Genetic correlation for H1xET47 family. The performance is computed as the Pearson correlation coefficients between genetic effect of two traits. The considered traits are the 18 Chlorophyll *a* fluorescence parameters and three target traits (i.e. leaf count (LC), tree height (TH) and trunk diameter (TD)), for treatment conditions 2, 3 and 4. Note that, the genetic effects were obtained based on SNP data for the corresponding family and using rrBLUP model for each trait.

	IBR	PI_{total}	φR_o	ΨE_o	φE_o	φP_o	$\frac{\varphi P_o}{(1-\varphi P_o)}$	$\frac{\delta R_o}{(1-\delta R_o)}$	$\frac{\Psi E_o}{(1-\Psi E_o)}$	$\frac{RC}{ABS}$	$\frac{RE_o}{RC}$	$\frac{DI_o}{RC}$	$\frac{ET_o}{RC}$	$\frac{TR_o}{RC}$	$\frac{ABS}{RC}$	F_o	F_M	$\frac{F_V}{F_M}$
**LC2**	-0.304	0.187	0.072	0.271	0.254	0.171	0.226	-0.176	0.222	0.275	-0.278	-0.217	-0.12	-0.284	-0.269	0.047	0.147	0.172
**TH2**	-0.2	0.144	0.131	0.184	0.21	0.005	-0.006	-0.032	0.193	0.217	-0.151	-0.126	-0.149	-0.229	-0.196	-0.01	-0.059	0.004
**TD2**	-0.158	0.186	0.166	0.124	0.107	0.09	0.108	0.07	0.102	0.196	-0.068	-0.107	-0.174	-0.175	-0.155	0.038	0.086	0.089
**LC3**	-0.12	0.093	-0.014	0.111	0.125	0.021	0.011	-0.099	0.125	0.18	-0.194	-0.076	-0.119	-0.151	-0.126	-0.051	-0.115	0.02
**TH3**	-0.226	0.125	0.099	0.223	0.284	-0.081	-0.07	-0.084	0.262	0.208	-0.141	-0.055	-0.042	-0.225	-0.164	0.068	-0.093	-0.081
**TD3**	-0.301	0.211	0.159	0.312	0.327	0.093	0.107	-0.153	0.28	0.335	-0.261	-0.193	-0.174	-0.358	-0.305	0.024	0.058	0.093
**LC4**	-0.184	0.244	0.139	0.311	0.315	0.12	0.123	-0.161	0.312	0.345	-0.302	-0.233	-0.232	-0.353	-0.318	-0.108	-0.012	0.119
**TH4**	-0.216	0.05	0.026	0.221	0.31	-0.152	-0.141	-0.159	0.284	0.171	-0.149	0.005	0.041	-0.207	-0.127	0.1	-0.14	-0.153
**TD4**	-0.139	0.108	0.143	0.195	0.231	-0.024	-0.014	-0.043	0.167	0.2	-0.102	-0.08	-0.071	-0.235	-0.18	0.122	0.057	-0.024
**LC2**	0.061	-0.031	-0.064	-0.109	-0.165	0.111	0.153	0.162	-0.173	-0.136	0.201	0.125	0.158	0.202	0.178	0.152	0.159	0.113
**TH2**	-0.017	0.07	0.113	0.105	0.105	0.052	0.051	0.018	0.113	-0.028	0.143	0.015	0.192	0.054	0.041	0.264	0.199	0.053
**TD2**	0.043	0.081	0.038	0.021	0.019	-0.013	0.014	0.005	0.025	0.025	0.008	0.03	0.003	0.014	0.02	0.131	0.041	-0.013
**LC3**	-0.096	0.003	0.005	0.02	-0.005	0.09	0.11	0.046	0.001	0.008	0.046	0.014	0.012	0.039	0.031	0.079	0.118	0.09
**TH3**	-0.095	0.059	0.094	0.108	0.106	0.069	0.059	-0.001	0.109	-0.035	0.137	0.006	0.199	0.057	0.04	0.294	0.176	0.07
**TD3**	0.099	-0.009	-0.03	-0.094	-0.125	0.01	0.04	0.19	-0.118	-0.096	0.181	0.151	0.102	0.178	0.171	0.286	0.16	0.011
**LC4**	-0.034	0.016	0.022	0.014	-0.003	0.058	0.072	0.08	-0.013	-0.007	0.094	0.043	0.066	0.066	0.059	0.116	0.125	0.058
**TH4**	-0.13	0.009	0.079	0.071	0.059	0.091	0.062	0.029	0.061	-0.109	0.213	0.025	0.282	0.112	0.083	0.336	0.204	0.093
**TD4**	-0.018	-0.074	-0.059	-0.036	-0.04	-0.003	-0.017	0.03	-0.043	-0.139	0.158	0.089	0.248	0.145	0.128	0.321	0.178	-0.002
**LC2**	0.128	-0.056	-0.12	0.085	0.09	-0.041	-0.06	-0.199	0.144	-0.066	-0.104	0.05	0.042	0.058	0.056	0.117	0.048	-0.039
**TH2**	0.175	-0.164	-0.185	-0.072	-0.072	-0.059	-0.062	-0.149	-0.05	-0.105	-0.125	0.088	0.004	0.092	0.093	0.124	0.073	-0.056
**TD2**	0.052	-0.121	-0.108	0.039	0.038	-0.028	-0.027	-0.164	0.035	-0.003	-0.177	0.012	-0.017	-0.012	-0.003	0.012	-0.034	-0.026
**LC3**	0.214	0.027	-0.051	0.119	0.114	-0.016	0.005	-0.16	0.173	-0.018	-0.08	0.05	0.032	0.042	0.046	0.183	0.189	-0.015
**TH3**	0.193	-0.179	-0.206	-0.048	-0.053	-0.041	-0.057	-0.186	-0.011	-0.112	-0.144	0.066	0.011	0.09	0.083	0.092	0.063	-0.037
**TD3**	0.122	-0.101	-0.063	0.073	0.049	0.045	0.028	-0.121	0.076	-0.04	-0.098	-0.022	0.002	0.016	0.002	0.021	0.093	0.048
**LC4**	0.226	-0.192	-0.211	-0.05	-0.048	-0.097	-0.091	-0.218	-0.014	-0.193	-0.071	0.174	0.084	0.204	0.197	0.322	0.226	-0.094
**TH4**	0.157	-0.205	-0.169	-0.064	-0.082	0.017	-0.021	-0.128	-0.04	-0.113	-0.075	0.031	0.014	0.099	0.076	0.088	0.124	0.022
**TD4**	-0.003	-0.091	-0.005	0.072	0.025	0.159	0.123	-0.052	0.057	0.015	-0.102	-0.115	-0.044	-0.035	-0.065	-0.061	0.158	0.162

Table T5: Genetic correlation for H1xG family. The performance is computed as the Pearson correlation coefficients between genetic effect of two traits. The considered traits are the 18 Chlorophyll *a* fluorescence parameters and three target traits (i.e. leaf count (LC), tree height (TH) and trunk diameter (TD)), for treatment conditions 2, 3 and 4. Note that, the genetic effects were obtained based on SNP data for the corresponding family and using rrBLUP model for each trait.

	IBR	PI_{total}	φR_o	ΨE_o	φE_o	φP_o	$\frac{\varphi P_o}{(1-\varphi P_o)}$	$\frac{\delta R_o}{(1-\delta R_o)}$	$\frac{\Psi E_o}{(1-\Psi E_o)}$	$\frac{RC}{ABS}$	$\frac{RE_o}{RC}$	$\frac{DI_o}{RC}$	$\frac{ET_o}{RC}$	$\frac{TR_o}{RC}$	$\frac{ABS}{RC}$	F_o	F_M	$\frac{F_V}{F_M}$
**LC2**	0.219	0.14	0.205	0.187	0.234	-0.031	0.021	0.025	0.228	0.241	-0.001	0.018	-0.026	-0.232	-0.057	0.034	0.05	-0.031
**TH2**	0.309	0.134	0.239	0.228	0.343	-0.097	-0.139	0.008	0.375	0.236	0.007	0.019	0.01	-0.227	-0.055	-0.007	-0.086	-0.096
**TD2**	0.186	0.106	0.212	0.235	0.29	0.039	0.035	-0.077	0.292	0.281	-0.092	-0.064	-0.057	-0.273	-0.14	-0.002	0.043	0.039
**LC3**	0.182	0.142	0.026	0.042	0.147	-0.26	-0.182	0.117	0.171	0.118	0.02	0.187	-0.012	-0.12	0.119	0.184	0.036	-0.26
**TH3**	0.291	0.138	0.232	0.237	0.35	-0.078	-0.137	-0.038	0.37	0.251	-0.032	-0.016	0	-0.255	-0.093	-0.024	-0.093	-0.077
**TD3**	0.148	0.129	0.24	0.188	0.25	-0.016	-0.025	0.071	0.253	0.209	0.046	0.019	-0.011	-0.236	-0.058	0.066	0.068	-0.016
**LC4**	0.201	0.241	0.226	0.183	0.24	-0.018	0.007	0.078	0.255	0.217	0.03	0.028	-0.014	-0.216	-0.044	0.065	0.079	-0.018
**TH4**	0.243	0.136	0.281	0.264	0.357	-0.011	-0.068	-0.014	0.363	0.259	-0.002	-0.069	-0.026	-0.278	-0.145	-0.083	-0.119	-0.011
**TD4**	0.2	0.16	0.334	0.31	0.362	0.122	0.039	-0.105	0.359	0.278	0.004	-0.162	0.047	-0.274	-0.222	-0.087	0.005	0.122
**LC2**	0.094	0.046	-0.009	-0.078	-0.107	0.034	0.07	0.147	-0.06	-0.084	0.068	0.06	-0.095	0.11	0.093	0.098	0.128	0.035
**TH2**	-0.058	0.114	0.086	0.051	0.073	-0.075	-0.036	0.078	0.096	0.028	0.124	0.076	0.03	0.004	0.033	0.062	-0.011	-0.074
**TD2**	-0.022	0.036	-0.014	-0.023	-0.02	-0.053	-0.019	-0.049	0.013	0	-0.058	0.063	-0.103	-0.003	0.023	0.074	0.019	-0.052
**LC3**	0.031	-0.002	-0.066	-0.081	-0.064	-0.139	-0.114	0.005	-0.013	-0.132	0.041	0.167	0.016	0.147	0.159	0.24	0.029	-0.138
**TH3**	0.007	0.13	0.094	0.048	0.078	-0.114	-0.058	0.106	0.11	0.032	0.135	0.105	0.01	-0.001	0.041	0.061	-0.044	-0.113
**TD3**	-0.053	0.142	0.118	0.08	0.081	0.042	0.062	0.083	0.101	0.118	0.004	-0.059	-0.143	-0.115	-0.096	-0.038	0.039	0.043
**LC4**	-0.068	0.156	0.125	0.108	0.127	-0.023	0.005	0.054	0.161	0.055	0.123	0.026	0.052	-0.026	-0.006	0.082	0.047	-0.022
**TH4**	0.032	0.137	0.089	0.034	0.048	-0.067	-0.015	0.108	0.072	0.08	0.037	0.055	-0.107	-0.053	-0.012	0.035	-0.012	-0.066
**TD4**	-0.048	0.171	0.166	0.095	0.096	0.019	0.045	0.106	0.107	0.116	0.113	-0.047	-0.059	-0.114	-0.09	-0.035	0.02	0.021
**LC2**	-0.03	0.011	-0.062	0.023	0.024	0.013	0.012	-0.16	-0.006	0.114	-0.249	-0.032	-0.325	-0.1	-0.073	-0.063	-0.023	0.013
**TH2**	-0.066	-0.139	-0.164	-0.028	0.026	-0.18	-0.229	-0.322	0.008	-0.027	-0.319	0.098	0.005	0.019	0.056	-0.02	-0.332	-0.18
**TD2**	-0.011	-0.103	-0.175	-0.063	-0.029	-0.146	-0.172	-0.275	-0.074	0.009	-0.354	0.09	-0.132	0.005	0.044	0.01	-0.25	-0.146
**LC3**	0.01	-0.016	-0.091	-0.046	-0.033	-0.079	-0.063	-0.154	-0.057	0.064	-0.273	0.067	-0.291	-0.021	0.022	0.023	-0.041	-0.079
**TH3**	-0.066	-0.148	-0.176	-0.062	-0.007	-0.209	-0.255	-0.3	-0.027	-0.034	-0.329	0.126	-0.04	0.034	0.078	0.014	-0.341	-0.209
**TD3**	0.042	-0.098	-0.171	-0.099	-0.076	-0.155	-0.173	-0.237	-0.101	-0.029	-0.32	0.122	-0.085	0.057	0.088	0.013	-0.271	-0.155
**LC4**	-0.092	-0.097	-0.125	-0.015	0.002	-0.076	-0.119	-0.262	-0.044	0.02	-0.305	0.032	-0.088	-0.02	0	-0.03	-0.192	-0.076
**TH4**	-0.095	-0.142	-0.184	-0.071	-0.021	-0.175	-0.239	-0.285	-0.039	-0.001	-0.384	0.076	-0.103	-0.011	0.03	-0.032	-0.351	-0.175
**TD4**	0.051	-0.117	-0.162	-0.062	-0.021	-0.192	-0.223	-0.286	-0.05	-0.014	-0.342	0.123	-0.015	0.016	0.065	0.025	-0.363	-0.192

■ **Table T6:** Selection performance of L_{21}-Joint, ridge regression (RR), multiple LASSO (mLASSO), elastic-net (EN), Bayesian LASSO (BL) and multi trait BayesB (mBayesB). The performance is computed as the proportion of correctly selected worse performing lines with respect to leaf count (LC), tree height (TH) and trunk diameter (TD). For populations H1xG (i.e. left panel) and H1xET47 (i.e. right panel), the assessment is conducted for genomic and phenomic predictions models accounting for environmental conditions. Numbers in bold represent the best performance and we write xx to express that, the corresponding statistical approach was not used for phenomic prediction.

**Selected proportion of worst performing lines**														
	**RR**	**Mlasso**	**EN**	**GBLUP**	**BL**	**MBayesB**	**L21-Joint**	**RR**	**Mlasso**	**EN**	**GBLUP**	**BL**	**MBayesB**	**L21-Joint**
**(A1): GP_H1xG**								**(A2): GP_H1xET47**						
LC	10	15	20	**25**	5	10	5	30	10	15	15	10	35	**40**
TH	15	15	15	**20**	10	5	15	20	15	20	15	15	30	**35**
TD	15	10	**20**	15	5	5	**20**	15	**25**	10	**25**	20	**25**	**25**
**(B1): PP_H1xG**								**(B2): PP_H1xET47**						
LC	5	15	20	xx	20	10	**30**	30	25	20	xx	30	20	**40**
TH	5	**15**	10	xx	**15**	10	10	25	30	**35**	xx	15	20	15
TD	15	10	30	xx	5	15	**35**	30	20	**35**	xx	20	**35**	25
